# Prevalence of HCV genotypes in district Mardan

**DOI:** 10.1186/1743-422X-10-90

**Published:** 2013-03-20

**Authors:** Suliman Qadir Afridi, Muhammad Nouman Zahid, Muhammad Zubair Shabbir, Zeenat Hussain, Nadia Mukhtar, Muhammad Yasin Tipu, Fareeha Akhtar, Tahir Yaqub

**Affiliations:** 1 Quality Operations Laboratory, UVAS, Lahore 54000, Pakistan; 2 University Diagnostic Laboratory, University of Veterinary and Animal Sciences, Lahore 54600, Pakistan; 3 Consultant Pathologist, The Medical Laboratories (Pvt), Lahore 54650, Pakistan

**Keywords:** Hepatitis, Hepatitis C virus, HCV genotypes, Prevalence, Pakistan

## Abstract

**Background:**

Approximately 170 million people are infected with Hepatitis C virus (HCV) worldwide. The prevalence of chronic HCV infections in Pakistan is about 5%, with most individuals being infected with HCV genotype 3a. Data on HCV genotypes distribution across various districts of the country are scarce. One example is district Mardan from where such data is available only from 17 individuals. Accordingly, the present study aimed at determining HCV genotypes distribution among 177 HCV RNA positive individuals from district Mardan.

**Findings:**

Serum samples (n = 215) from patients suspected of hepatitis C were collected and processed for Nested PCR based detection and subsequent genotyping. Gender-wise and age-wise differences in HCV prevalence and HCV genotypes distribution were determined by *χ*2 test. Out of the total 215 serum samples, 177 were found to be positive for HCV RNA. The genotype 3a was the most predominant genotype among HCV RNA positive samples with a prevalence of 90.3%, followed by genotype 1a (5.6%), mixed genotypes (2.8%), genotype 3b (0.6%) and genotype 4 (0.6%). The HCV prevalence was higher in young individuals than old people and was indicative of reduced survival rate beyond 40 years.

**Conclusion:**

HCV genotype 3a is the most predominant genotype in district Mardan. The state of the art preventive and therapeutic strategies should be implemented to control the spread of HCV infections. Further temporal studies involving different geographical areas of Pakistan, are required to improve the control measures for HCV infection.

## Findings

Hepatitis C is an infectious disease caused by Hepatitis C virus (HCV). HCV has a major impact on public health with over 170 million infected individuals. It has been considered to cause 25% of hepatocellular carcinoma (HCC) and 27% of cirrhosis cases all over the world [1]. Death rate due to HCV infection is very high and approximately 350, 000 people die every year after being infected with HCV. It is thought that HCV is 10 times more infectious than human immunodeficiency virus (HIV) [2]. Toxicity, resistance and cost of the treatment limit current therapy of HCV. To date, no vaccine or immunotherapy is available.

HCV is an enveloped positive-strand RNA virus. It belongs to the genus hepacivirus that is part of Flaviviridae family. HCV virion size is about 55–65 nm in diameter [3]. HCV genome (approximately 9.6 kb) encodes a polyprotein of about 3,010 amino acids, which is flanked at the 5'- and 3'- ends by small highly structured untranslated regions (UTR). This polyprotein precursor is cleaved by viral and cellular proteases, giving rise to 10 mature structural and nonstructural proteins. Core, E1 and E2 are structural proteins while a small hydrophobic peptide p7 separates the structural proteins from nonstructural proteins (NS). The nonstructural proteins include NS2, NS3, NS4A, NS4B, NS5A, and NS5B [4]. HCV envelope glycoprotein E1 and E2 are important for viral entry. HCV core protein interacts with lipid droplets [5] while p7 has been shown to participate in assembly and release of HCV particles [6]. NS2 has been shown to interact with envelope glycoproteins, p7 and NS3 and recruits viral proteins to lipid droplets [7]. NS3 protein plays a role in the recruitment of NS5A to lipid droplets and in the assembly of viral particles [8]. The formation of membranous web is the important function of NS4B [9]. The interaction of NS5A and apolipoprotein (ApoE) is required for the assembly and export of infectious virions [10]. NS5B is a RNA-dependent RNA polymerase (Dry) that is a critical enzyme for HCV RNA replication.

HCV shows high genetic variability and therefore, it is divided into six major genotypes and multiple subtypes. HCV has heterogeneous geographical distribution. HCV genotypes 4, 5 and 6 are restricted to more precise geographic regions (i.e., South Africa and Southeast Asia, Egypt, and Africa, correspondingly), whereas genotypes 1, 2, and 3 have a global distribution. In addition, being limited to different geographical areas these six genotypes have their own distinctive pattern of disease development and response to therapy.

In Pakistan, prevalence of HCV is 4.7% [11]. One of the major reasons for chronic HCV infection is lack of awareness of the disease and regular blood analysis, so most of the individuals remain ignorant about their status of HCV infection. A little has been reported about HCV and its genotypes prevalence in Pakistan. Therefore, this work has been conducted to determine baseline data on the prevalence of HCV genotypes in a district in North of Pakistan, the Mardan. The baseline information will serve in better understanding of infection, awareness in the public and subsequent control strategies.

The study was approved by Ethical Committee Bacteriologist to Government of Punjab, Health Department (*No. 677/Bact.*). From hundreds of blood samples referred from district Mardan to The Medical Laboratory, Lahore for the detection and genotyping of HCV RNA, a total of 215 blood samples were randomly selected to detect and genotype HCV RNA. The age of clinically suspected hepatitis C patients was between 10–50 years. Of these 111 were males and 104 were females. Serum from each blood sample was separated at 3000 g for 5 min, labeled and stored at −20°C until used within a week. RNA was extracted using Qiagen kit (Invitrogen, Corp., California; USA) as per manufacturer’s instructions. Complimentary DNA (cDNA) was synthesized using Invitrogen kit as per manufacturer’s instructions. Briefly, 10 μL of extracted RNA was incubated at 37°C for 60 min along with gene-specific reverse primer and 200U of Moloney Murine Leukemia Virus (M-MuLV) reverse transcriptase. The cDNA was amplified through Nested PCR.

Different HCV genotypes were detected by using allele-specific primers reported by Ohno et al. [12]. Temperature profile of PCR machine was adjusted at 94°C for 5 min for initial denaturation, followed by 35 cycles, each of 45 s denaturation at 94°C, 45 s annealing at 62°C and 45 s extension at 72°C, with final extension at 72°C for 5 min. The final PCR product obtained either after qualitative or genotype-specific PCR was subjected to electrophoresis in 2% agarose gel containing ethidium bromide along with 100-bp DNA ladder (Invitrogen, Corp., California; USA). The banding pattern, to determine genotype specific bands, was photographed in Gel Doc System. The prevalence and distribution of each genotype among different age groups and sex was analyzed by Chi-square statistics implemented in SPSS version 16 for windows (IBM Corporation, 2008). *P* value < 0.05 was considered significant.

**Figure 1:**
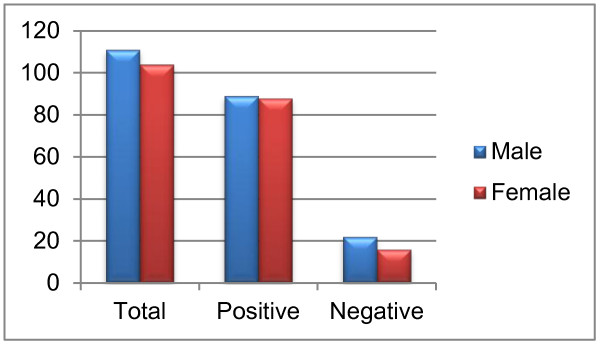
Prevalence of HCV RNA positive samples.

**Figure 2:**
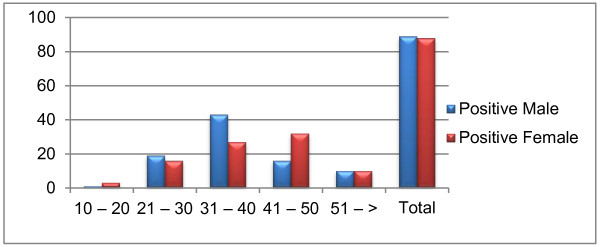
Prevalence of HCV RNA positive samples in different age groups.

Of the total 215 sera, only 177 were positive for HCV RNA, with almost equal representation from males and females (males = 89 (50%) and females = 88 (49%); *P* > 0.05; Figure 1). Serum samples were categorized into different age groups such as 10–20 years, 21–30 years, 31–40 years, 41–50 years and above 50 years, and percentage of HCV RNA positive samples was determined. The prevalence of HCV positive samples was 80%, 75%, 80%, 87% and 95% for age groups 10–20 years, 21–30 years, 31–40 years, 41–50 years, and above 50 years, respectively. There was no statistically significant difference in number of HCV RNA positive samples between different age groups (*P* > 0.05). The percentage of male HCV RNA positive samples was higher in age group 31–40 years while most of HCV RNA positive samples in age group 41–50 years were from females (*P* < 0.05) (Figure 2).

**Figure 3:**
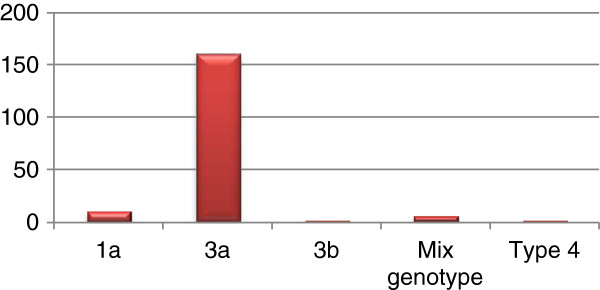
Prevalence of different HCV genotypes in Mardan.

Genotype 3a was found to be the most prevalent genotype (160/177 = 90.3%), followed by genotypes 1a (10/177 = 5.6%), 3b (1/177 = 0.6%), 4 (1/177 = 0.6%) and mixed genotype (5/177 = 2.8%). Thus, genotype 3a was significantly more abundant than 1a, 3b, type 4, and mixed genotypes (*P* < 0.05) (Figure 3).

The prevalence of different HCV genotypes was also stratified according to age groups 10–20 years, 21–30 years, 31–40 years, 41–50 years, and above 50 years and is provided in Table 1. While genotype 3a was predominant in all age groups, a high prevalence of genotype 1a and mixed genotypes was observed in age group 41–50 years.

**Table 1:** Prevalence of HCV genotypes in different age groups

**Age group**	**Genotypes**						
	**3a**	**1a**	**3b**	**Type 4**	**Mixed genotype**	**Total**	**%age**
10–20	3	1	0	0	0	4	2.2%
21–30	35	0	0	0	0	35	19.7
31–40	64	3	0	0	2	69	38.9
41–50	38	5	1	0	3	47	26.5
51 - >	20	1	0	1	0	22	12.4
Total	160	10	1	1	5	177	100

Mardan is located in the southwest of province Khyber Pakhtunkhwa (KPK). Urban proportion of the district is 20.2% whereas 79.8% is the rural proportion. A report conducted by Tehsils & Unions in the district Mardan, Government of Pakistan has mentioned that compared to urban areas HCV prevalence is more in rural areas. Therefore, out of 215 sera, 50 were taken from urban areas whereas 165 sera were taken from rural areas.

Prevalence of HCV genotypes has been reported from different areas of Pakistan. Inamullah et al. [13] reported genotype 3a (34.1%) as the most prevalent genotype in district Swat followed by genotypes 2a (8.1%), 3b (7%), 1a (5.4%) and mixed genotype (7.6%). Ahmad et al. [14] also found 49.5% prevalence of genotype 3a in district Swat. Ali et al. [15] processed 415 HCV RNA positive patients for genotyping from Khyber Pakhtunkhwa province and found 57.83% prevalence of genotype 3a, followed by 6.2% of genotype 3b. In a study conducted in Lahore, Ahmad et al. [16] found 55.9% prevalence of genotype 3a and 3.2% of genotype 3b. Overall, it has been shown that genotype 3a is 62% prevalent among HCV positive samples in Pakistan while prevalence of 3b, 1a, 2a and mixed genotypes is 9%, 3%, 2.144% and 4.718%, respectively [17]. In our study, we have also observed that in 41–50 years age group, females have high prevalence of HCV than males while this is reverse in 31–40 years age group where males are more affected than females. A total of 40 HCV seropositive samples belonging to seven different locations of Baluchistan were studied by Afridi et al. [18] and genotype 3a was the most prevalent genotype among all samples.

When a comparative study was made among different cities and districts of Pakistan including district Mardan, genotype 3a was found to be the most common genotype [17]. In this study, we have also compared the prevalence of HCV among different age groups. We found that there was no specificity of HCV dissemination among different age groups and all groups showed closely related prevalence of HCV. Moreover, we observed difference of HCV prevalence in males and females among different age groups. The age group between 31–40 years showed that males of this group are at high risk of having HCV infection while females of age group between 41–50 years displayed high HCV infection rate than males. It will be interesting to find out the factors involved in this gender specification.

It is concluded that genotype 3a is the most prevalent HCV genotype in Mardan. The higher prevalence of HCV genotype 3a all around the Pakistan suggests that Pakistani population is more susceptible to genotype 3a as compared to other genotypes. Further studies on the prevalence of HCV genotypes in different areas of Pakistan involving large geography and population are needed which will further help to strengthen and revise preventive as well as therapeutic strategies throughout the country.

## Competing interests

The authors declare that they have no competing interests.

## Authors’ contribution

MTY, MNZ and MZS contributed in study design and manuscript write up. ZH^3^ provided lab facilities and contributed reagents. SQA did perform the work whereas NM, FA and YT did analyze data and supervised the study. All authors read and approved the final manuscript.
